# Supplementary material for: Robust Immune Response and Protection against Lethal Pneumococcal Challenge with a Recombinant BCG-PspA-PdT Prime/Boost Scheme Administered to Neonatal Mice
Source: Vaccines (Basel). 2024 Jan 25;12(2):122. doi: 10.3390/vaccines12020122 (PMC10893189; doi:10.3390/vaccines12020122)
Supplement: Supplementary file 1 [file vaccines-12-00122-s001.zip › vaccines-2752141-supplementary.pdf]

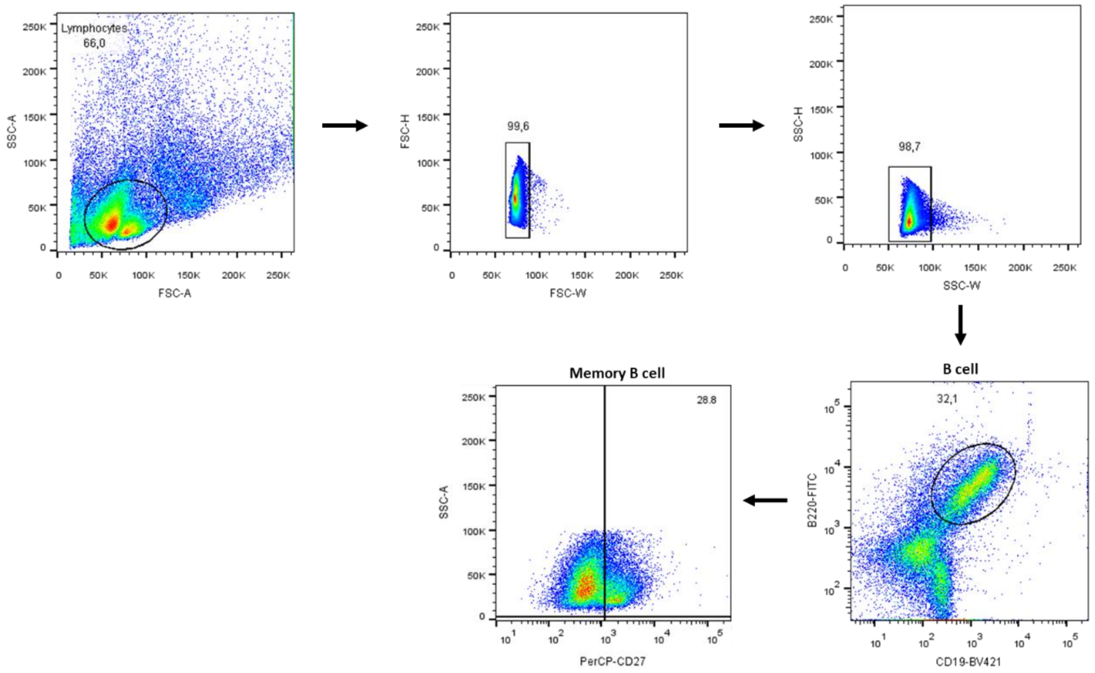

Figure S1. Gating strategy to identify B cells and Memory B cells. Spleen cell suspensions were gated for lymphocytes by FSC (Forward scatter) and SSC (Side scatter) and then live/dead cells were stained with FVS (Fixable Viability Stains, BD Horizon™). The B cells were gated from the live cells and were identified as B220+CD19+, and Memory B cells, B220+CD19+CD27+.

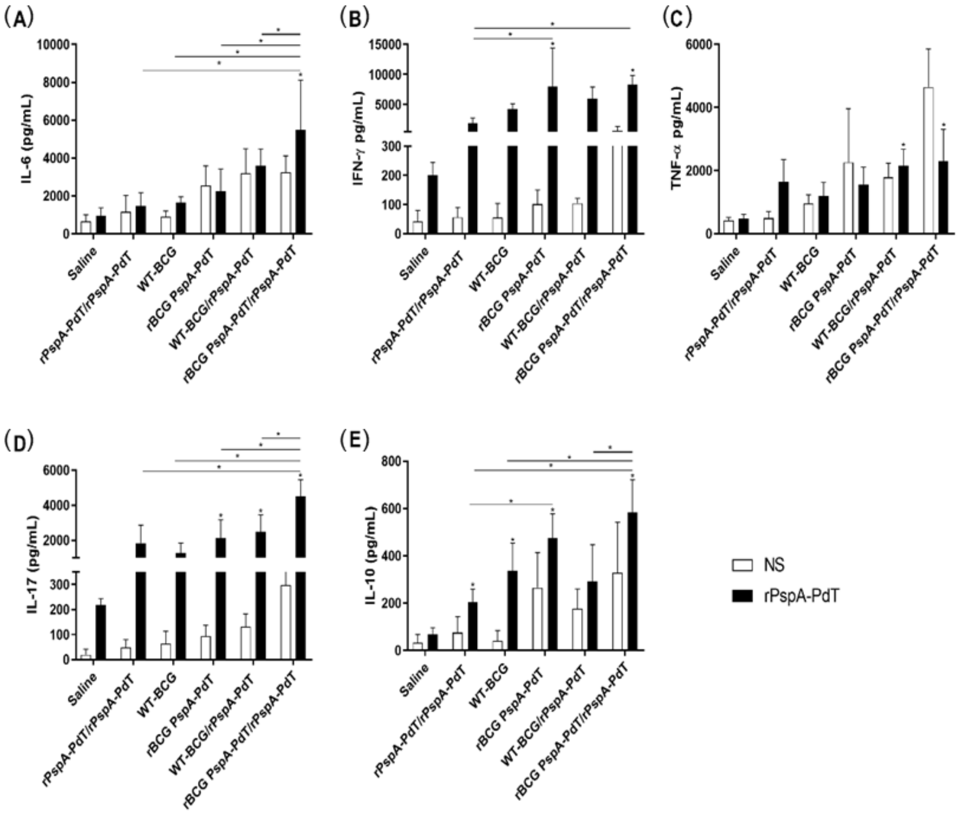

Figure S2. Prime/boost with rBCG PspA-PdT/PspA-PdT induces inflammatory cytokines in the lungs in neonatal mouse model. Lung cells from immunized mice 9 days after the booster dose were cultured for 48 h in the absence (medium only) or presence of rPspA-PdT as stimulus. Cytokine levels were measured by mouse Th1/Th2/Th17 cytometric bead array (CBA). Cytokine levels of IL-6 (A), TNF-α (B), IFN-γ(C), IL-17 (D) and IL-10 (E) are displayed. All results are represented by the means ± SD. Statistical differences were determined by one-way ANOVA with a Bonferroni test. *p values ≤ 0.05 were considered statistically significant. Asterisks over the columns refer to the comparison with the infection group.

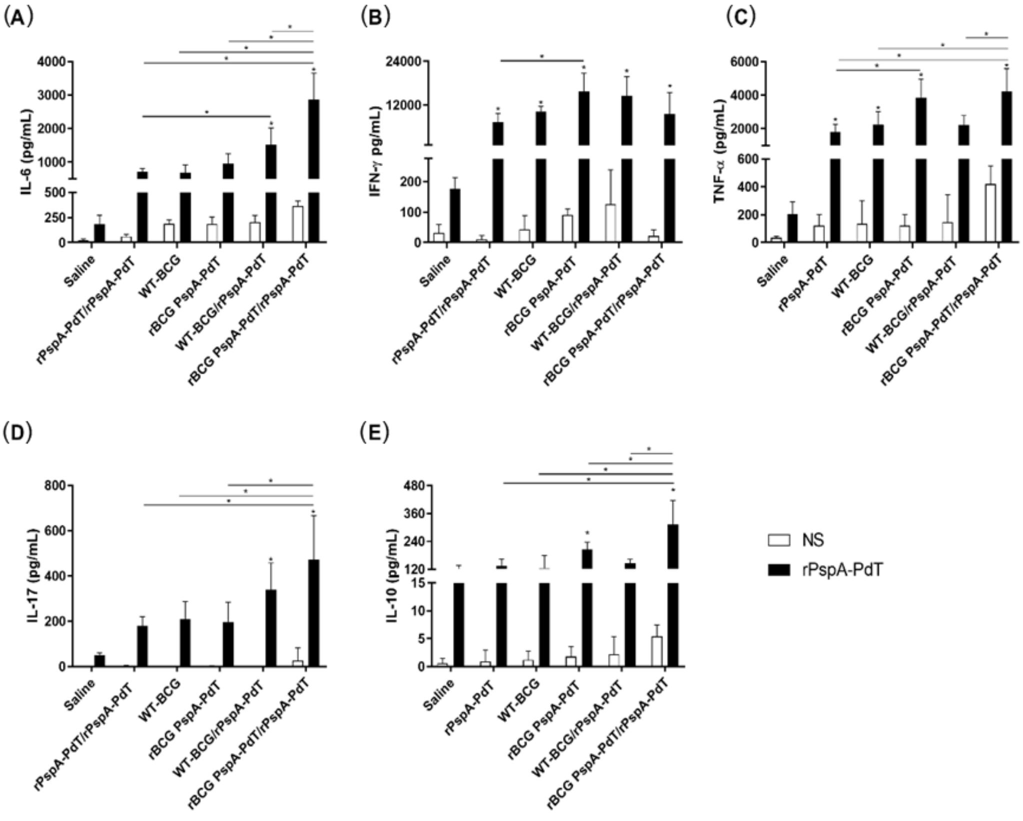

Figure S3. Prime/boost with rBCG PspA-PdT/PspA-PdT induces inflammatory cytokines in the spleens in the neonatal mouse model. Spleen cells from immunized mice 9 days after the booster dose were cultured for 48 h in the absence (medium only) or presence of rPspA-PdT as stimulus. Cytokine levels were measured by mouse Th1/Th2/Th17 cytometric bead array (CBA). Cytokine levels of IL-6 (A), TNF-α (B), IFN-γ(C), IL-17 (D) and IL-10 (E) are displayed. All results are represented by the means ± SD. Statistical differences were determined by one-way ANOVA with a Bonferroni test. *p values ≤ 0.05 were considered statistically significant. Asterisks over the columns refer to the comparison with the infection group.

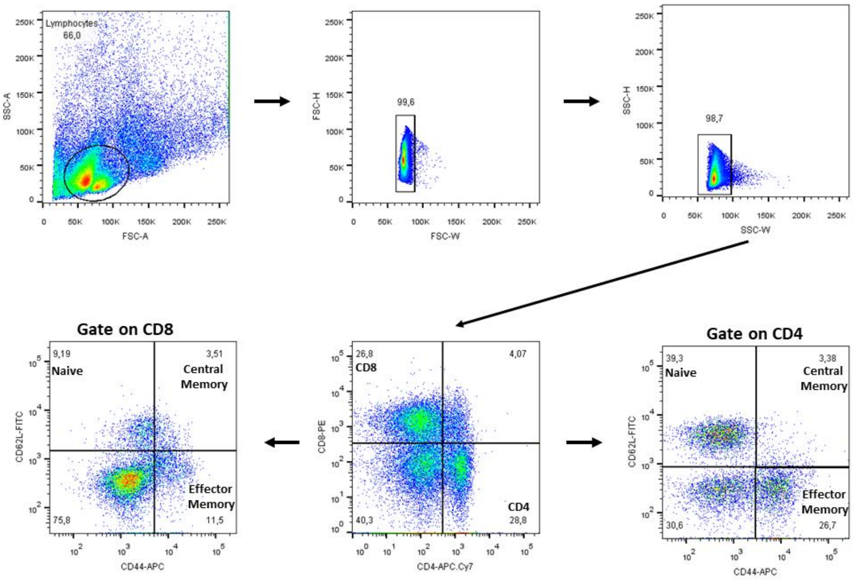

Figure S4. Gating strategy to identify subsets of memory T cells. Spleen cell suspensions were gated for lymphocytes by FSC (Forward scatter) and SSC (Side scatter) and then live/dead cells were stained with FVS (Fixable Viability Stains, BD Horizon™). The CD4+ and CD8+ cells were gated from the live cells and then the different subsets of T CD4+ and CD8+ cells were identified as: Memory central T cells (CD62L+ CD44+) and Effector T cells (CD62L- CD44+).
